# Long-term groundwater protection efficiency of different types of sanitary landfills: Data description

**DOI:** 10.1016/j.dib.2019.104488

**Published:** 2019-09-09

**Authors:** Igor Madon, Darko Drev, Jakob Likar

**Affiliations:** a Komunalno stanovanjska družba Ajdovščina, Goriška 23B, 5270, Ajdovščina, Slovenia; b Faculty of Civil Engineering and Geodesy, University of Ljubljana, Slovenia; c Faculty of Natural Sciences and Engineering, University of Ljubljana, Slovenia

**Keywords:** Risk assessment, Sanitary landfill, Aquifer pollution

## Abstract

Data presented in this paper are related to the research article “Long-term risk assessments comparing environmental performance of different types of sanitary landfills'’ (Madon et al., 2019). Overall environmental risks were quantitatively assessed by calculating probabilities that an assumed aquifer lying directly below the landfill of a particular type will be polluted due to landfill-derived impacts as long as the pollution potential referring to each of the four types which were compared exists. A specific model was built for the purpose, described in the companion MethodX article (Madon et al., 2019). Uncertainty was taken in consideration by attributing input parameters required for modeling with probability distributions. When loosely defined groups of landfills are to be compared, which was the objective of the related research article, these distributions can be nothing but approximate and spread out, however, the values tend to cluster together around the averages which are characteristic for particular landfill types. Secondary data from scientific literature were mostly used to estimate probability density functions for the inputs, however, when referring to one of the four landfill types which were compared, primary data were used as well. The resultant outputs derived by running Monte Carlo simulations are given as time dependent variables. In this article, probability distributions for the outputs are graphically presented comparing environmental performance of different landfill types.

**Table:** Specifications Table

Subject	Environmental science
Specific subject area	Waste disposal, Groundwater protection, Quantitative risk assessment, Comparative risk analysis
Type of data	Figures Tables
How data were acquired	Input data were acquired by - filtering and preprocessing secondary data obtained by means of scientific data mining (referring to 3 types of sanitary landfills) - performing long-term research and monitoring at a relatively small, full scale waste disposal and recycling site (referring to one sanitary landfill type) Output data were acquired by - simulation of input data using specific risk assessment model built within the @Risk software environment, product of Palisade Corporation
Data format	referring to the inputs to the model: - preprocessed secondary data - primary raw data referring to the outputs from the model: - simulated data derived from the input data mentioned above
Parameters for data collection	Secondary data were collected from the selected peer reviewed articles where reasonably well defined systems were studied, however, vagualy defined information derived from large number of sources was also taken into account when considering spread of possible values. Primary raw data were collected at the Ajdovščina waste disposal and recycling site performing environmental monitoring which lasted long enough for establishing long-term waste stabilization trends.
Description of data collection	When using @RISK software program, uncertain input variables are entered as probability distribution functions in cell formulas. Data used to construct these best-fit input probability curves were mainly collected from (1) specific peer reviewed studies where raw data derived from large number of landfills of a particular type have already been processed for different purposes and/or (2) from primary raw data. Output data were derived by performing Monte Carlo simulations utilizing above mentioned probability distributions for the inputs. Distribution of possible outcomes is obtained by letting a computer recalculate the worksheet repeatedly, each time using different randomly selected sets of values.
Data source location	for raw secondary data: - global sources (data related to 3 landfill types) for raw primary data: - Ajdovščina low-cost waste disposal and recycling site, Slovenia (data related to one landfill type) for processed input and output data: - KSD Ajdovščina, Slovenia
Data accessibility	With this article.
Related research article	Igor Madon, Darko DREV, Jakob LIKAR Long-term assessments comparing environmental performance of different types of sanitary landfills Waste Management https://doi.org/10.1016/j.wasman.2019.07.001

**Table:** 

**Value of the Data** The acquired simulated data– • can be valuable when uncertainty is appropriately acknowledged, but misleading when not • could be of interest for local waste-management developers in low-income countries who want to upgrade their dumpsites • can be helpful for hydrogeologists who perform long-term environmental risk assessments for already closed- or new landfill sites • can provide landfill operators with new ideas before upgrading or closing their facilities • can be used as thought-provoking material for landfill designers and regulators

## 1 Data

Compilation of all of the possible modeling outputs is presented in Table 1.

**Table 1:** Compilation of outputs obtained by simulation.

	Outputs	Units
C_t_	Concentration of a specific pollutant within the leachate at the bottom of the landfill	Concentration (probability distribution of values for the selected post-closure year)
Q_t_	Yearly leachate losses into the subsoil	Volume (probability distribution of values for the selected post-closure year)
QRP_t_	Yearly release of a specific pollutant into the subsoil (''quantity of a reference pollutant'')	Mass (probability distribution of values for the selected post-closure year)
QRP_max_	Maximal quantity of a (reference) pollutant annually discharged into the aquifer (i.e., emissions during the post-closure year when the emitted quantity appears to be the largest: QRP_t_ = QRP_max_)	Mass (probability distribution of values for the most polluting post-closure year)
CUMQRP_max_	Quantity of reference pollutant cumulatively emitted into the subsoil considering overall life span until the landfill of a certain type exhibits pollution potential for causing moderate level of aquifer pollution.	Mass (probability distribution of values)
MLP_starting_	Moderate level of aquifer contamination - commencement of the unfolding event	Required number of post-closure years for the ''event'' to happen (probability distribution of values)
MLP_ending_	Moderate level of aquifer contamination - cessation of the unfolding event	Required number of post-closure years for the ''event'' to happen (probability distribution of values)
SLP_starting_	Severe level of aquifer contamination - commencement of the unfolding event	Required number of post-closure years for the ''event'' to happen (probability distribution of values)
SLP_ending_	Severe level of aquifer contamination -cessation of the unfolding event	Required number of post-closure years for the ''event'' to happen (probability distribution of values)
ILP_starting_	Irreversible level of aquifer pollution - commencement of the event	Required number of post-closure years for the ''event'' to happen (probability distribution of values)
P_MLP_	Probability for MLP to happen considering overall life span until the landfill of a certain type exhibits pollution potential for causing moderate level of aquifer pollution.	Probability (discrete value) P_MLP_ = MLP_starting max_ = MLP_ending max_
P_SLP_	Probability for SLP to happen considering overall life span until the landfill of a certain type exhibits pollution potential for causing severe level of aquifer pollution.	Probability (discrete value) P_SLP_ = SLP_starting max_ = SLP_ending max_
P_ILP_	Probability for ILP to happen considering overall life span until the landfill of a certain type exhibits pollution potential for causing ''irreversible'' level of aquifer pollution.	Probability (discrete value) P_ILP_ = ILP_starting max_

**Fig. 1:**
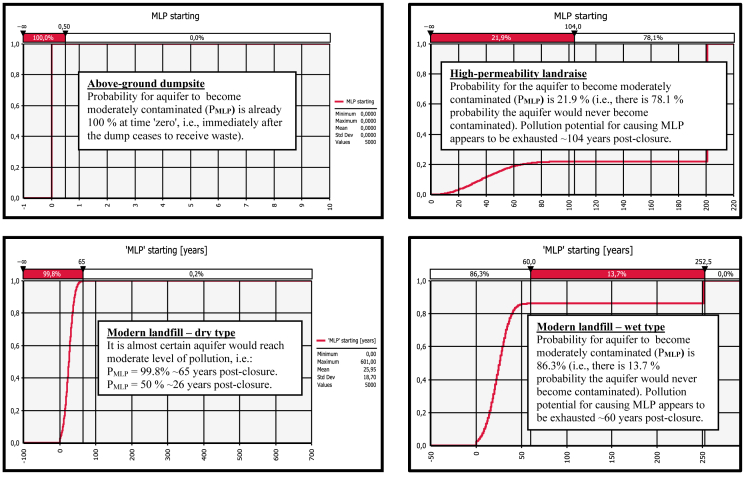
Derived comparative graphs for the output "moderate level of aquifer pollution – commencement of the unfolding event".

**Fig. 2:**
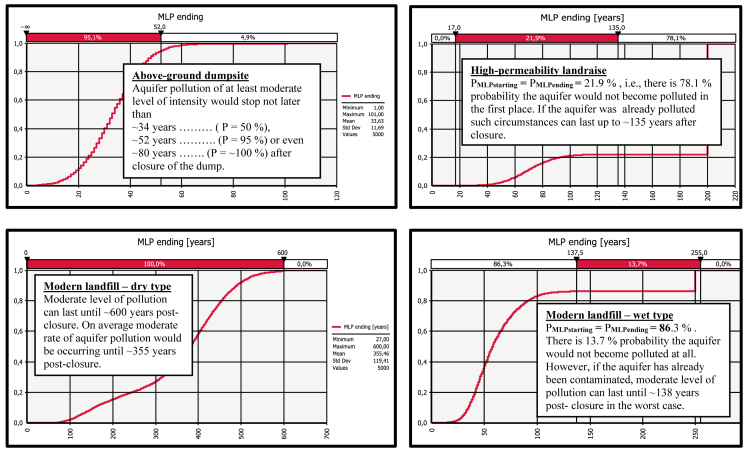
Derived comparative graphs for the output "moderate level of aquifer pollution – cessation of the unfolding event".

**Fig. 3:**
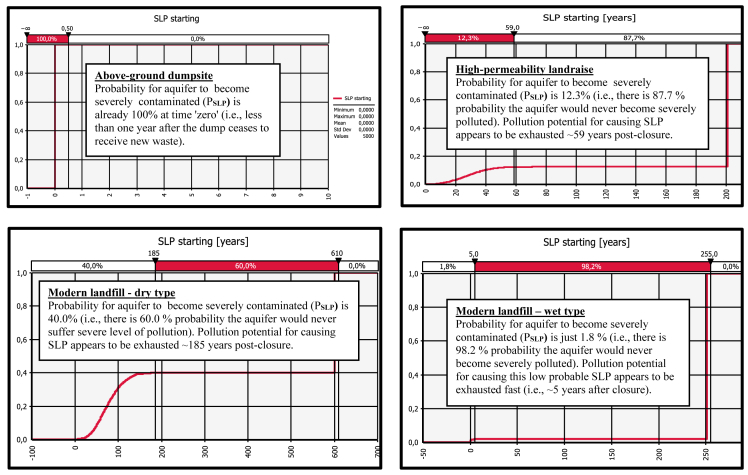
Derived comparative graphs for the output "severe level of aquifer pollution – commencement of the unfolding event".

**Fig. 4:**
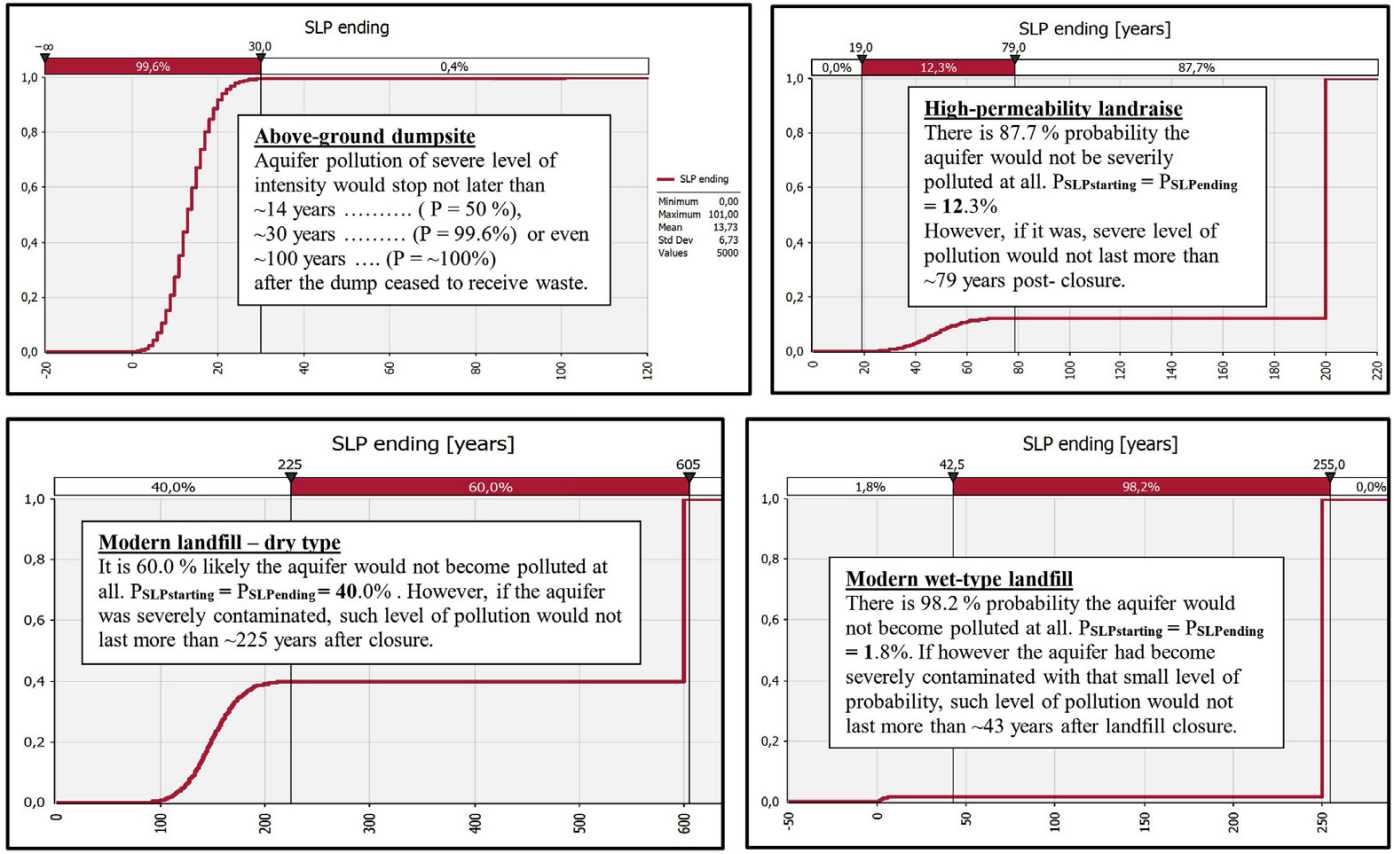
Derived comparative graphs for the output "severe level of aquifer pollution – cessation of the unfolding event".

**Fig. 5:**
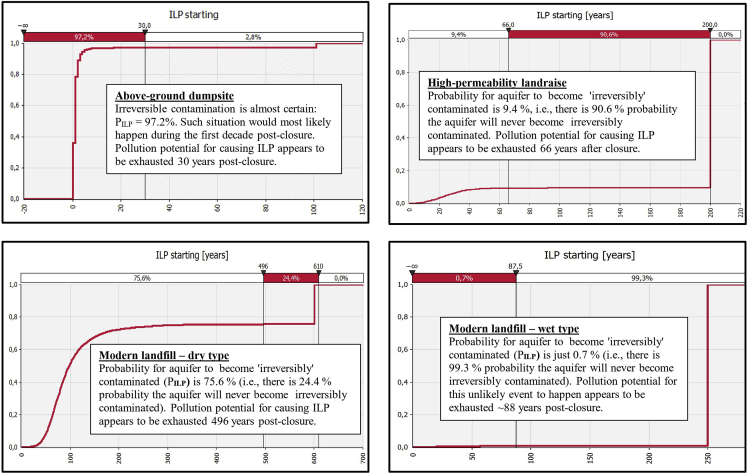
Derived comparative graphs for the output "irreversible level of aquifer pollution – commencement of the unfolding event".

**Fig. 6:**
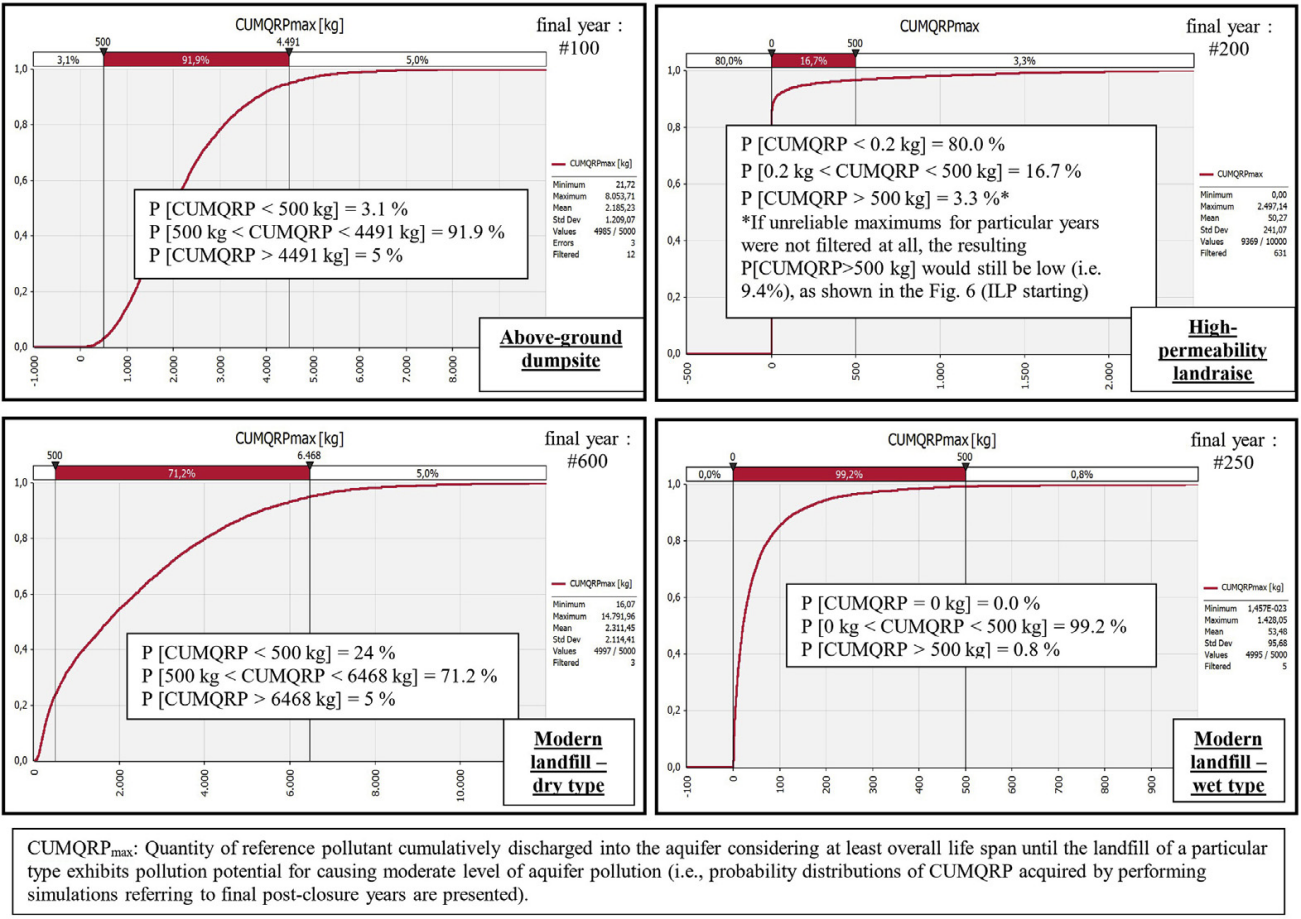
Derived comparative graphs for the output "quantity of reference pollutant cumulatively discharged into the aquifer".

Resultant graphs for the outputs "MLP_starting_", "MLP_ending_'', ''SLP_starting_", "SLP_ending_'', ''ILP_starting_" and "CUMQRP_max_'' are presented in Fig. 1, Fig. 2, Fig. 3, Fig. 4, Fig. 5, Fig. 6. Each figure consists of 4 graphs supplied with explanation text in order the differences in environmental performance between particular landfill types (i.e., above-ground dumpsite, high-permeability landraise, modern dry-type landfill and modern wet-type landfill, respectively) can be more easily seen.

**Fig. 7:**
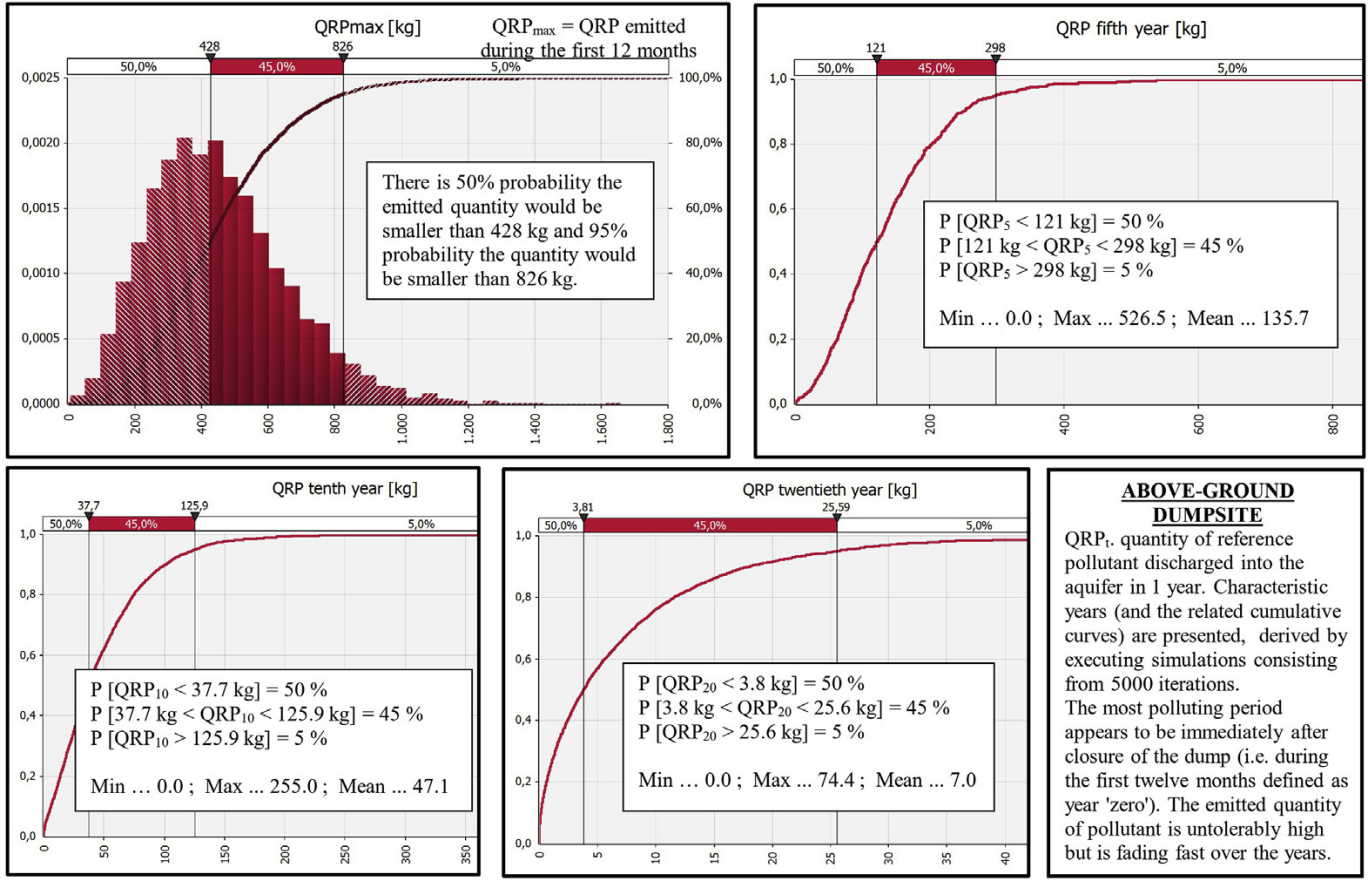
Derived comparative graphs for the output "quantity of reference pollutant discharged into the aquifer" referring to above-ground dumpsites characteristic post-closure years.

**Fig. 8:**
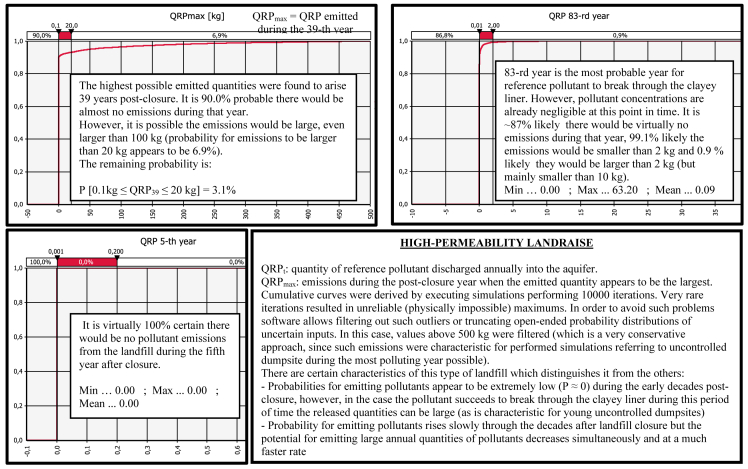
Derived graphs for the output "quantity of reference pollutant discharged into the aquifer" referring to high-permeability landraises characteristic post-closure years.

**Fig. 9:**
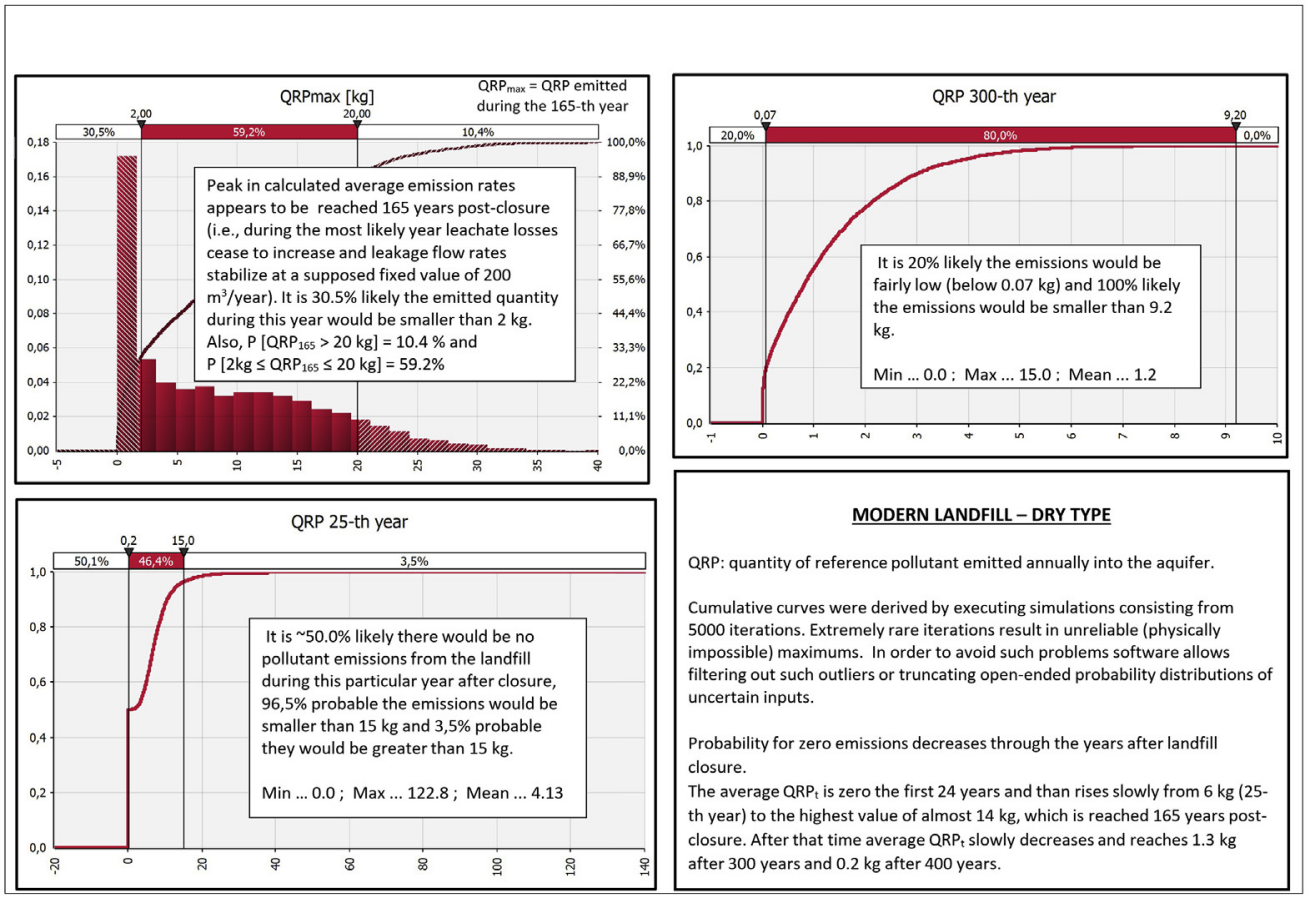
Derived graphs for the output "quantity of reference pollutant discharged into the aquifer" referring to modern landfills of dry type characteristic post-closure years.

**Fig. 10:**
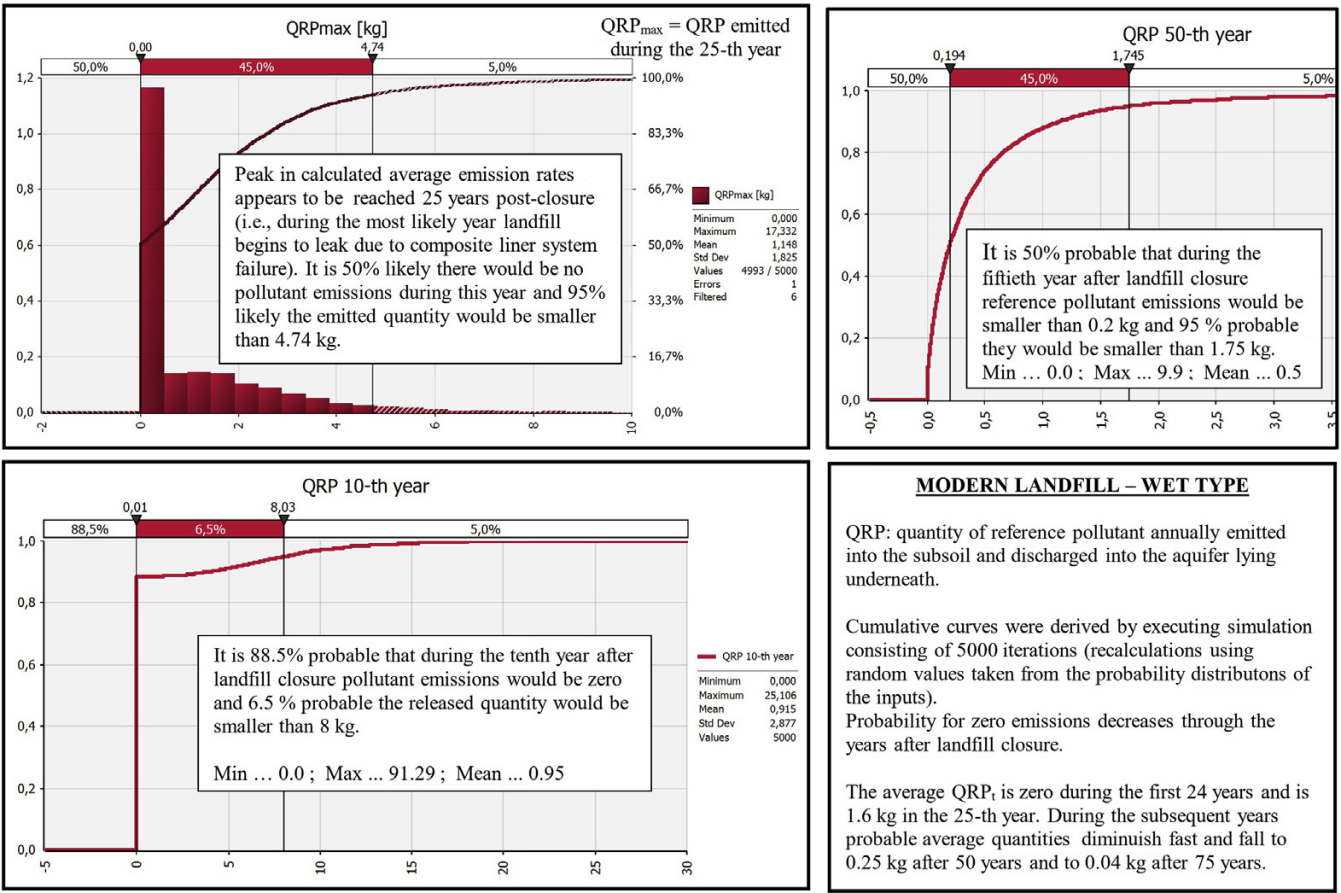
Derived graphs for the output "quantity of reference pollutant discharged into the aquifer" referring to modern landfills of wet type characteristic post-closure years.

Comparative graphs for the output ‘‘QRP_t_’’ are presented in Fig. 7, Fig. 8, Fig. 9, Fig. 10 demonstrating ‘‘QRP’’ during the characteristic post-closure years (which includes particular post-closure years during which ‘‘QRP_max_’’ is reached). Each figure refers to one of the four antagonistic types of landfills which were compared.

Sensitivity analises for parameters "QRP_max_" and "CUMQRP_max_'' are provided as a supplementary material (Appendix-A).

## 2 Experimental design, materials, and methods

Waste disposal is one of those industries that generate data of considerable variety, veracity, and variability. These properties make secondary data analysis a big problem for the researchers in the field. Information describing hydrogeological configuration of landfill sites is usually missing or is deficient. Eventual leakages into the subsoil are usually undetected. It is therefore not surprising that no programs or code files for filtering and analyzing raw data from secondary sources exist that can be used to find reasonable correlations between environmental performances of landfills on one side and variables which contribute to these performances on the other side. This kind of approach is intrinsically unfit anyway, because too little post-closure time has expired so far in order to observe long-term groundwater protection effectiveness of most objects which can be hystorically described as modern landfills.

According to the model which was developed [1], [2], there are just two decisive quantities which have to be known in order to perform long-term risk assessments from landfills: 1) primary leachate losses into the subsoil "Q_t_" and 2) concentration of pollutants in primary leachate at the bottom of the landfill ‘"C_t_". Both of these parameters generally change over time after landfill closure. They already represent quantities on the output side of the model. Important point however is that these outputs can be obtained by simulation modeling utilizing rather small number of input variables which can be convincingly attributed with probability density functions processing already available data and information. Simulated data for parameters ‘‘Q_t_’’ and ‘‘C_t_’’ are needed to derive many other, more complex outputs, however, once the model is established, all of the outputs are acquired in a single simulation step. Flowchart demonstrating the applied concept is presented in the related MethodX article [2].

Probability distributions for the inputs can be directly fitted to already available raw data from secondary and/or primary sources when such data exist. However, this is usually not the case. All direct and indirect information which is available has to be used to construct an input model instead. The goal is to obtain an approximation that captures the key characteristics of the underlying input process. @Risk program software [3] includes vast assortiment of probability functions which can be readily used to attribute input variables with estimated probability distributions. Several options for fitting distributions to raw data are available as well.

A common hydrogeological and hydrological setting was set up for modeling purposes in order to compare environmental performance of different landfill types. This was done in a way that the expected differences between the four antagonistic landfill types would show up as clearly as possible. The presumed common setting is described below:

- an aquifer exists immediately below the landfill subgrade, separated only by a thin vadose zone
- the local terrain is semipermeable (including the above mentioned vadose zone)
- the landfill is placed in a humid region.

Input variables needed for performing model simulations are compiled below:

- C_0_ [mg/L]: initial concentration of the reference pollutant immediately after landfill closure
- T_0.5_ [years]: half-life period characterizing reference pollutant concentration decline within the leachate at the bottom of the landfill
- t_failure_ [years]: post-closure time which has to pass for composite liner system to fail
- q_0_ [liters per hectare per day [lphd]]: initial specific leachate losses into the underground soon after the liner has failed
- T_2_ [years]: time needed for leachate losses to double after the system fails
- q_max_ [lphd]: maximal possible leachate losses into the subsoil per unit area of landfill footprint
- k_sat_ [m/s]: hydraulic conductivity coefficient of a bottom clay liner
- d [m]: bottom clay liner (or natural clay stratum) thickness
- Q_precip_ [mm]: annual precipitation
- p_undg_ [%]: part of annual precipitation which is infiltrated into the landfill generating landfill leachate, but only that portion which percolates further down to the aquifer

Only parameters required to calculate pollutant concentrations "C_t_" (i.e.,"C_0_" and "T_0.5_") are invariably involved as modeling inputs for all landfill types. Other parameters, i.e. those which are required to calculate leakages "Q_t_" appear to be rather specific for particular landfill types, i.e.:

- when referring to dry- and wet-type modern landfills, leachate losses into the subsoil are considered to be a stochastic phenomenon; "t_failure_", "q_0_","T_2_" and "q_max_" are the related variables needed for performing simulations of long-term leakages
- hydraulic system at the bottom of a high-permeability landraise (HPL) type of landfill is however deterministic; leakages are calculated using the Darcy law; nevertheless, the required inputs "k_sat_" and "d" are considered to be variables not just due to uncertainties which exist when dealing with permeability measurements of small specimens in laboratory, etc., but to consider the expected diversity among the landfills of this type when comparing landfill types as groups
- "Q_precip_" and "p_undg_" are exclusively used to simulate annual leakages emanating from the uncontained landfills (dumpsites); leachate losses from contained landfills are only indirectly related to local hydrologic and hydrogeologic factors

Input variables were quantifyed as described below:

1. Probability density functions for parameters "K_sat_" and "d" were selected according to the characteristics which define HPL as a landfill type.
2. Probability density functions for parameters "Q_precip_" and "p_undg_" were selected according to the characteristics of the presumed common hydrogeological and hydrological setting.
3. Probability distribution-estimations for the inputs "C_0_" and "T_0.5_" were mostly acquired by processing large amounts of secondary data which are only indirectly related to the parameters "C_0_" and "T_0.5_". When referring to HPL, raw primary data were used for the purpose instead. The main sources are presented below:
  - Laner [4] for modern landfills
  - Kjeldsen and Christophersen [5] for dumpsites
  - Madon [6] for high permeability landraises

Specific approaches and techniques were occassionally used to obtain the desired information, which would be otherwise unattainable.

4. Leakage rate from modern landfills is considered to be zero until the post-closure time when bottom liner system fails. Pivato [7] constructed failure probability curve based on groundwater monitoring data from 30 landfill sites in northern Italy. Distribution of "t_failure_" values appeared to be approximately normal with average time approximately 25 years and standard deviation approximately 12.5 years. This density distribution was attributed to parameter "t_failure_" to build an input model.
5. Typical leakage rates from modern landfills occuring immediately after the bottom liner fails appear to be very low. According to the measurements performed on double-lined landfills (Geoservices Inc., [8], EPA/600/R-02/099, [9], Moo-Young et al., [10]), frequencies of leakage-rates ranges appear to be distributed as follows (in liters per hectare per day)
  • 0 lphd (few cases)
  • 0–10 lphd (most of cases)
  • 10–100 lphd (a lot of cases)
  • 100–1000 lphd (few cases)
  • 1410 lphd (one case)

Measurement 1410 lphd most probably represents the case where clay liner functioned as a sole element of waste containment system, i.e., as if geomembrane has not existed due to some major failure. This value could be a good estimate for "q_max_".

Generally, buried HDPE geomembranes have an estimated service life that is measured in terms of hundreds of years. The three stages of degradation and approximate associated durations for each as obtained from the laboratory testing program described in the report [9], are: (i) antioxidant depletion (≈200 years), (ii) induction (≈20 years), and (iii) half-life (50% degradation) of an engineering property (≈750 years). Therefore, with ageing, geomembranes deteriorate by definition and eventual leakages on average slowly increase.

The inputs were attributed with values as described below:

• Initial leakages "q_0_" were chosen to fall mainly within the 0–10 lphd range (mean value = 4.4 lphd, st.dev. = 1.4 lphd)
• Leakage rates were considered to increase slowly through the decades (average doubling time "T_2_" was set to be 30 years and st. deviation also 30 years)
• Maximal leakage "q_max_" could have also been attributed with a probability distribution function in order to include highest possible leakage rates which were already measured (such as those higher than 1000 lphd), however, a discrete cut-off value of 110 lphd was used instead in order to be somewhat complaisant to conventional dry-type landfills comparing them to others when running simulations

Simulated data referring to average leachate flow rates into the subsoil during the characteristic post-closure years comparing performances of different landfill types are shown in Table 2. Simultaneously occuring values for reference-pollutant- concentrations in primary leachate are presented, too.

**Table 2:** Leachate fugitive flow rates into the subsoil and simultaneously occuring ref. pollutant average concentrations within the primary leachate (average values derived from simulations).

Post-closure year	Mean values for Q_t_ and C_t_ variables	Above-ground dumpsite	High-permeability landfill (HPL)	Modern dry-type landfill	Modern wet-type landfill
10th	Q_t_ [lphd] [mm/year]	1233 45	426 15.6	0 0	0 0
	C_t_ [mg/L]	20	(62)	1009	446
50th	Q_t_ [lphd] [mm/year]	1233 45	426 15.6	7.8 0.3	15.6 0.6
	C_t_ [mg/L]	<1	(<1)	505	8
100th	Q_t_ [lphd] [mm/year]	1233 45	426 15.6	24.6 0.9	49.2 1.8
	C_t_ [mg/L]	<1	<1	221	<1
200th	Q_t_ [lphd] [mm/year]	1233 45	426 15.6	109.6 (max value) 4 (max value)	219.2 (max value) 8 (max value)
	C_t_ [mg/L]	<1	<1	38	<1

Note that -

1.) flow through the clayey liner underneath the HPL's does not result into emissions until the pollutants penetrate the liner and break through on its bottom side (until this happens, reference pollutant concentration values within the leachate at the bottom of the landfill are shown in parentheses).

2.) upperbound (max) leachate rates of flow into the subsoil are reached 165 years after closure on average for modern landfills according to simulation results.

3.) water losses from the landfill into the subsoil "Q_t_" are expressed in liters per ha per day as well as in units commonly used to describe amount of precipitation (mm per year).

4.) leakage rates from above-ground dumpsites situated in humid climates are generally high (however, pollutant concentrations within the primary leachate are generally low and decline rapidly after landfill closure).

5.) leakage rates from modern landfills are generally very low or even non-existent (but pollutant concentrations within the primary leachate are generally high and decline very slowly in the case of dry-type landfills).

The applied methodology, including the approaches used to derive probability distribution estimates for input variables is more thoroughly described in the companion MethodX article [2].
